# Differences in distress: Variance and production of American Crocodile (*Crocodylus acutus*) distress calls in Belize

**DOI:** 10.1002/ece3.6556

**Published:** 2020-08-25

**Authors:** Miriam Boucher, Marisa Tellez, James T. Anderson

**Affiliations:** ^1^ School of Natural Resources West Virginia University Morgantown WV USA; ^2^ Crocodile Research Coalition Stann Creek Belize; ^3^ Marine Science Institute University of California Santa Barbara Santa Barbara CA USA

**Keywords:** American Crocodile, Belize, bioacoustics, crocodylian, sound

## Abstract

Acoustic communication of American Crocodiles (*Crocodylus acutus*) is relatively understudied. Our overall aim was to determine the acoustic structure of wild American Crocodile distress calls, distinguish call differences among size classes (hatchling, juvenile, sub‐adult, and adult), and investigate call production on a gradient of human disturbance. American Crocodile distress calls have strong frequency modulation and are comprised of multiple harmonics in a downsweeping pattern. Measured parameters (total duration, first quartile duration, maximal frequency, first quartile frequency, end frequency, slope of first quartile, slope of last quartiles) differed significantly among size classes (*p* < .05). Hatchling distress calls are higher in frequency and strongly modulated, whereas calls produced by sub‐adults and adults showed little modulation, are lower in frequency, and have greater overall duration. Proportion of crocodiles that produced distress calls during capture differed by size class and sampling location, particularly adult distress calls which are reported here to be produced with undocumented frequency. We determined that American Crocodiles of all size classes produce distress calls at varying rates among study sites. Our results demonstrate that American crocodiles produce distress call more frequently at sites with higher anthropogenic activity. Measured call parameters of juveniles and hatchling American crocodiles also varied among sites in relation to human disturbance. Calls recorded at sites of high anthropogenic impact have increased duration and less modulation which may adversely affect response to emitted distress calls. Proportional and call parameter variances suggest anthropogenic activity as a driver for increased call production and alteration of call parameters at high human‐impacted sites.

## Graphical abstract

We determined the acoustic structure of wild American Crocodile distress calls upon capture and evaluated differences among size classes and along a gradient of human disturbance. Calls varied among ages and were higher at the most disturbed site. Acoustic ecology has important implications for assessing the health of crocodiles and the systems upon which they depend.

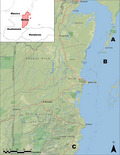

## 1 INTRODUCTION

Social interactions between crocodylians are facilitated through a variety of acoustic and physical signals. Of the acoustic signals produced, distress calls are perhaps the most frequently recorded call. Distress calls are repetitive chirps with multiple harmonics and frequency modulation (Garrick & Lang, 1977; Vergne, Pritz, & Mathevon, 2009). The distress call is commonly produced by juveniles as a warning to other crocodylians and also elicits a defense response from conspecifics (Britton, 2001; Campbell, 1973; Chabert et al., 2015; Herzog & Burghardt, 1977; Vergne, Aubin, Taylor, & Mathevon, 2011; Vergne et al., 2009). Production of this behaviorally significant call decreases as the size of the crocodylian increases in conjunction with reduced risk of predation (Staton, 1978). Although reported, production of distress calls by sub‐adult and adult crocodylians is rare (Staton, 1978). There is a paucity of data regarding the structure, production, and significance of distress calls among crocodylians, such as the American Crocodile (*Crocodylus acutus*), and the exploration is still in its infancy.

**FIGURE 1:**
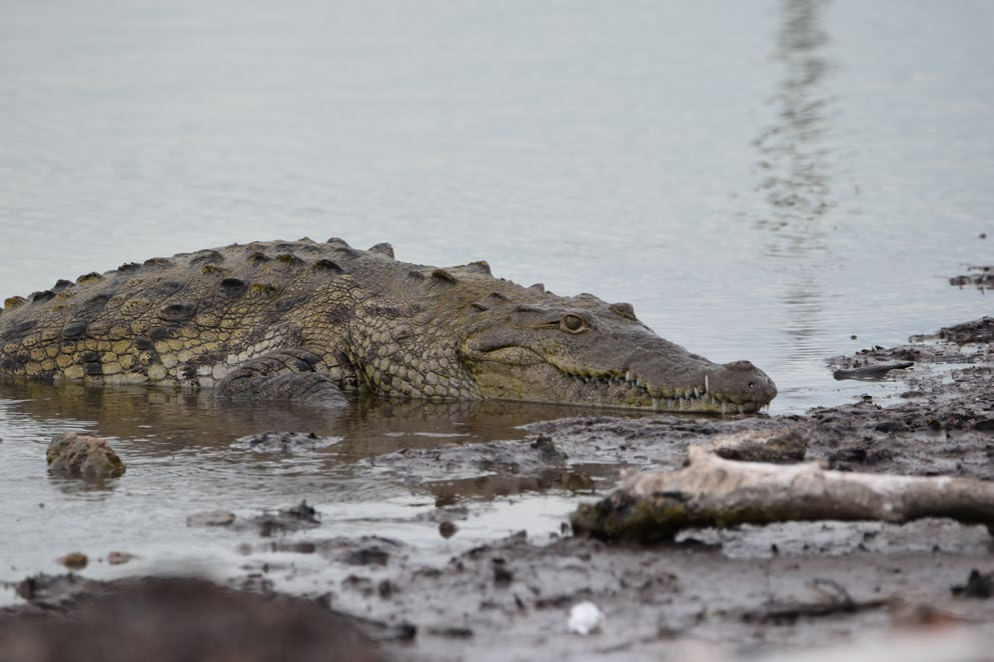
An adult American Crocodile photographed at one of the highly human‐impacted study sites sampled for acoustic recording on Ambergris Caye, Belize

American Crocodiles are the most widely distributed crocodylian in the New World, inhabiting coastal and lowland wetlands from southern Florida, USA, to northern South America (Ernst, Ross, & Ross, 1999; Platt & Thorbjarnarson, 2000). Although now a protected species in all of its range, previous exploitation in the mid‐1900s led to extirpation and declines of the American Crocodile in many areas. Populations drastically decreased in Belize between 1930 and 1970 (Platt & Thorbjarnarson, 2000; Rainwater & Platt, 2009). Although the commercial hunting that caused population declines has ceased, new threats have emerged putting recovering populations at risk. Degradation and diminishment of habitat as a result of widespread pollution and coastal development are significant threats to the American Crocodile in Belize (Platt, Rainwater, & Nichols, 2004; Platt & Thorbjarnarson, 2000; Rainwater, 2008). Ongoing research in various crocodilyan habitats of Belize is providing increasing evidence of the connection between environmental toxicity and mortality and/or morbidity currently observed in crocodiles (Tellez, unpubl. data). It is likely, however, that there are also ecological consequences of human influence on American Crocodiles in Belize (Figure 1). Additionally, there has been little dedicated study of American Crocodile bioacoustics anywhere in its range. Increasingly, anthropogenic noise and impact in wildlife habitat is demonstrated to have deleterious effects on wildlife acoustics and ecology (Blickley & Patricelli, 2010; Hildebrand, 2005; Laiolo, 2010). As an acoustically communicative species, it is feasible that American Crocodile sound production may be affected by anthropogenic disturbance. Herein we describe the acoustic structure of American Crocodile distress calls and explore a possible link between distress call production and anthropogenic impact.

## 2 MATERIALS AND METHODS

### 2.1 Study areas

**FIGURE 2:**
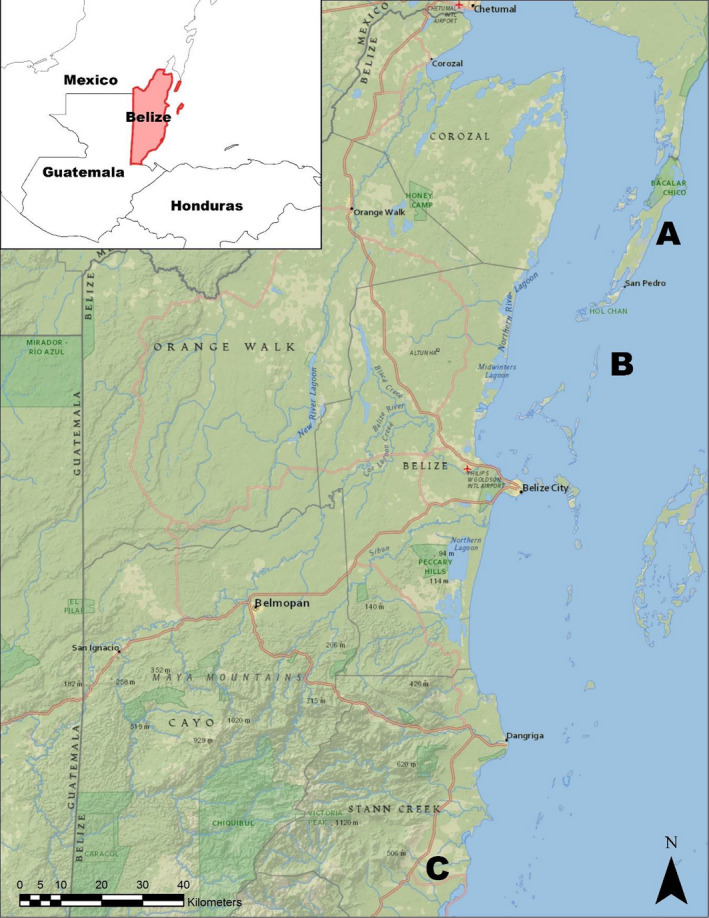
Belize, Central America, located on the eastern Caribbean seaboard. Letters dictate study area location; (A) Ambergris Caye, (B) Caye Caulker, and (C) Belize Aquaculture Limited

Belize is a small Mesoamerican nation (22,965 km^2^) paralleled by the Mesoamerican Barrier Reef and located south of Mexico on the eastern seaboard (Figure 2). We collected acoustic recordings in the coastal zone of Belize at Ambergris Caye, Caye Caulker, and Belize Aquaculture Limited west of the Placencia Peninsula.

Ambergris Caye is the southern extension of the Mexican Yucatan Peninsula separated from Mexico by a small channel. We collected American Crocodile recordings throughout the southern portion of the island in proximity to the highly developed San Pedro town center. We recorded captured crocodiles throughout the study area and in particular from the Coco Beach Resort lagoon, San Pedro sewage treatment ponds, and the Mahogany Bay Village development site.

Our second study area, Caye Caulker, is an offshore island located 8 km south of Ambergris Caye. American Crocodiles are distributed along the entire island and we attained recordings opportunistically throughout the study area during concurrent population surveys.

Belize Aquaculture Limited (BAL), our final study area, is a large commercial shrimp farm (3,642 ha) located approximately 8.3 km east of the northern tip of the Placencia peninsula. We recorded crocodiles captured in the effluent polishing lagoon and canal system located on the margins of the farming operation.

### 2.2 Wetland impact assessment

Concurrent to the collection of behavioral observations, we assessed study sites using a 6‐point scale (Table 1) to characterize human disturbance in the wetlands (Maine Department of Environmental Protection, 2013). Our assessments scored presence and severity of hydrologic modifications to the wetland, vegetative modifications to the wetland, evidence of pollutants, and acoustic disturbance and human contact. We used rankings of each category of stressor to determine an overall level of human disturbance for each study site.

**TABLE 1:** Description of stressor severity ranks for wetland human disturbance assessment (Maine Department of Environmental Protection, 2013)

Severity of stressor	Severity description	Rank
Not observed or unknown	Stressor is not observed or has no detrimental impact	0
Observed; minimal disturbance	Stressor is present and appears to have negligible impacts on wetland	1
Low disturbance	Stressor is present and appears to have minor impacts on wetland condition	2
Moderate disturbance	Stressor is present and appears to moderately impact wetland condition	3
High disturbance	Stressor is present and appears to significantly impact wetland condition	4
Severe disturbance	Stressor is present and appears to have major impacts on wetland condition	5

### 2.3 Capture techniques

As distress calls are produced when crocodiles are under duress or perceived threat, we collected recordings during capture of wild American Crocodiles. Crocodiles were captured as part of ongoing behavioral research and population surveys. The West Virginia University Animal Care and Use Committee (Protocol # 15‐0703) and the Belize Forest Department (BFD) approved the capture protocol. The lead author acquired research permits from the BFD prior to initiating any capture or recording (Ref. No. CD/60/3/15(45)). We captured and restrained crocodiles using conventional techniques detailed by Webb and Messel (1977). We captured smaller crocodiles by hand and larger animals by noosing or a treble hook. We did not secure the snout of crocodiles prior to recording to avoid altering distress call emission. Following the collection of recordings, or if the crocodile ceased to vocalize, we secured the jaws using electrical tape and applied eye coverings to reduce capture stress. We collected morphometric measurements from all captured crocodiles following the protocol of Webb and Messel (1978). We used the total length (TL) to classify captured crocodiles as hatchlings (TL < 35 cm), juveniles (TL = 36–90 cm), sub‐adults (TL = 91–180 cm), or adults (TL > 181 cm). Following the completion of health assessment and morphometric measurements, we released crocodiles at site of capture.

### 2.4 Acoustic recording

We recorded American Crocodiles on Ambergris Caye from May–August 2015, December 2015–January 2016, and March of 2016. Concurrent with ongoing population surveys, we collected recordings on Caye Caulker in January, March, and August 2016. We collected acoustic recordings from BAL June–August 2016. We recorded distress calls ad libitum in the field during capture events as recording conditions and difficulty of capture determined the actual number of recordings collected. Independent of acoustic recording, we noted incidence of call production for each individual capture as not all captures resulted in call production or successful recording. We collected distress call recordings from May 2015–January 2016 using a Marantz PDM661 or Roland R‐26 digital recorder coupled with a Senheiser ME67 shotgun directional microphone. During the March–August 2016 field seasons, we employed a Sony Zoom H5 digital recorder with an XY modular microphone capsule. We recorded all calls in.wav format at a sampling rate of 44.1 kHz and 24 bits per sample. Although equipment differed, sampling protocol remained consistent. We held hatchling and juvenile crocodiles in hand and collected recordings approximately 50 cm from the microphone. We recorded sub‐adult and adult crocodiles from 1–2 m from the microphone to ensure safety of personnel. We also recorded any calls or responses from nearby conspecifics, noting size class if possible, when we recorded distress calls of captured crocodiles.

### 2.5 Sound analysis

**FIGURE 3:**
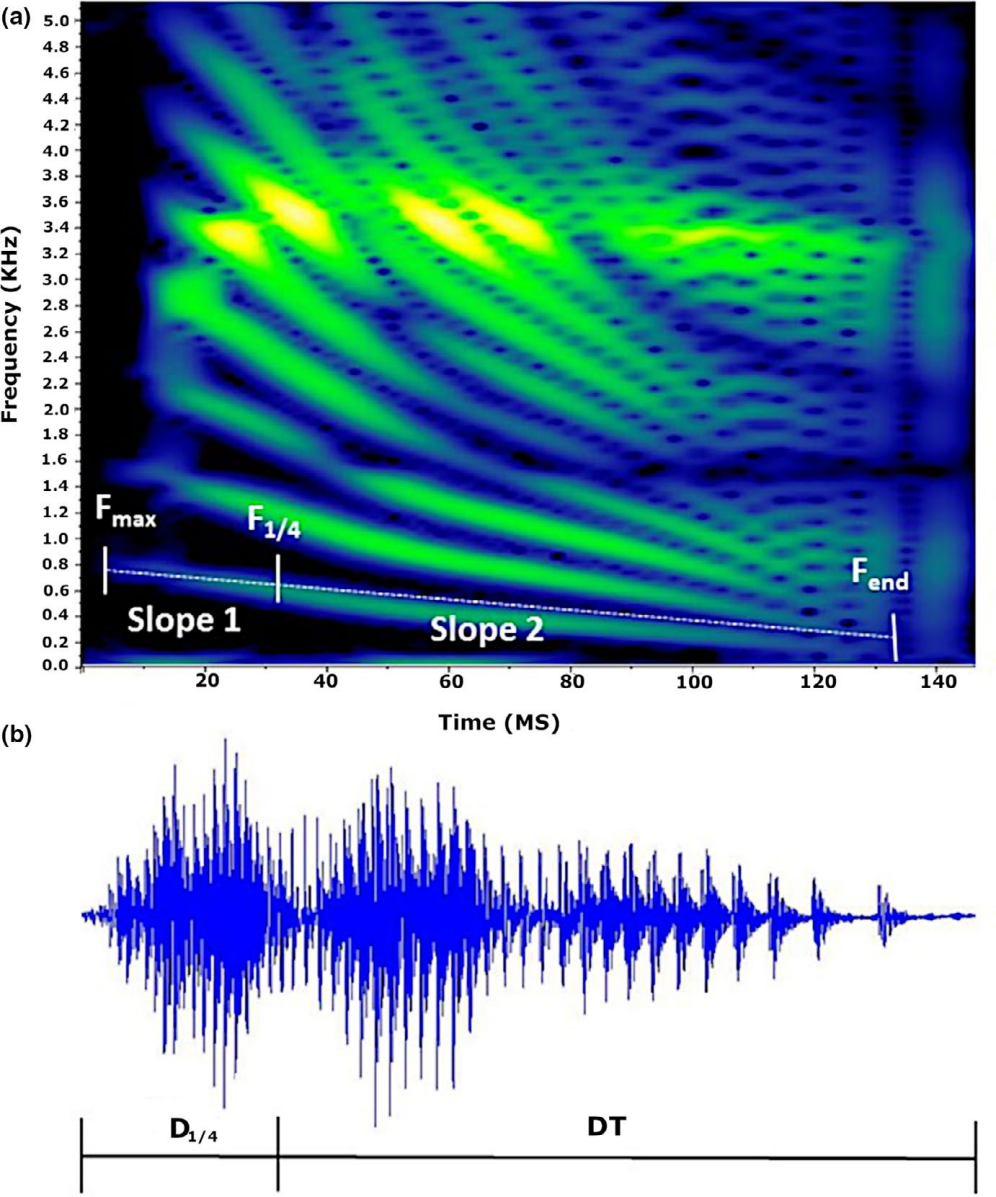
Spectrogram (a) and oscillogram (b) of a hatchling American Crocodile distress call, Ambergris Caye, Belize, 2016. Frequency parameter measurements, maximal fundamental frequency (*F*_max_), fundamental frequency at one‐quarter duration (*F*_1/4_), and frequency of the fundamental at the call end (*F*_end_) shown as derived from spectrographic analysis. Temporal properties, total duration (DT), and duration of first quartile (*D*_1/4_) shown from the measurement of distress call oscillogram

We performed acoustic analysis to determine the structure of distress calls for each size class. We analyzed five calls per individual, in one case only 3 calls were analyzed due to heavy background noise, and measured seven acoustic variables, two temporal and five spectral, using Raven Pro 1.5 acoustic analysis software (Bioacoustics Research Program, 2014). We used spectrographic analysis (window size 1,024, overlap 80%) of the fundamental frequency to determine maximal frequency (*F*_max_, Hz), frequency at end of first quartile (*F*_1/4_, Hz), and final frequency (*F*_end_, Hz; Figure 3a). Using call oscillograms, we measured temporal properties for total duration (DT, s) and duration of the first quartile (*D*_1/4_, s; Figure 3b). Measurement windows were drawn around the fundamental frequency and the maximal frequency values at the beginning, end, and first quartile were recorded. We used frequency and temporal measurements to calculate call modulation of the first temporal quartile slope (Slope 1, Hz/s, calculated as (*F*_1/4_ − *F*_max_)/*D*_1/4_), and the slope of the remaining three temporal quartiles (Slope 2, Hz/s, calculated as (*F*_end_ − *F*_1/4_)/(DT − *D*_1/4_)) (Vergne, Aubin, Martin, & Mathevon, 2012). Concurrent to call measurements, we recorded number of calls produced by each individual for 10‐, 20‐, and 30‐s intervals as total recording time varied between individual crocodiles. We began call counts at the first recorded call for each individual. We used size designation to organize and analyze distress call recordings by overall size class.

We performed statistical analyses using RStudio version 0.99.902 (RStudio Team, 2015). Our call parameter data did not meet the assumptions of normality or homogeneity of variance (*p* < .05) (Boucher, 2017). Thus, we analyzed call parameter means (DT, *D*_1/4_, *F*_max_, *F*_1/4_, *F*_end_, Slope 1, Slope 2) among size classes (hatchling, juvenile, sub‐adult, adult) by performing nonparametric Kruskal–Wallis (*H*) tests. We used Mann–Whitney post hoc testing with Bonferroni correction to determine pairwise variance of call parameters between size classes following a significant Kruskal–Wallis test (*p* < .05). We tested size class differentiation by call parameters using a principal component analysis cross‐validated by discriminant function analysis which assigned each recorded individual to a size class based on call parameters. To determine variance in number of calls produced (10‐, 20‐, 30‐s intervals), we performed one‐way analysis of variance (ANOVA) tests as these data met assumptions of normality (*p* > .05) and equality of variance (*p* > .05).

We analyzed differences in call production by determining the total number of American Crocodiles captured throughout the study for each location and compared them by size class. To account for small sample size, we aggregated data for Caye Caulker and BAL sites. We performed a 2‐sample test for equality of proportions with continuity correction (Newcombe, 2008) on total call production proportions among size classes. Designed for small sample sizes (<5), we performed Fisher's exact test (Agresti, 2002) to determine inequality in call production by size classes between Ambergris Caye, and the combined Caye Caulker and BAL sites. As a compliment to proportional tests, we performed Mann–Whitney tests to determine variance of spectral parameters of hatchling and juvenile calls between Ambergris Caye and aggregated Caye Caulker and BAL sites.

## 3 RESULTS

### 3.1 Wetland impact assessment

Ambergris Caye had the highest degree of human disturbance (rank = 3–4; *x̄* = 3.7; *SE* = 0.19) due to extensive habitat modification, pollution, and contact with humans. Moreover, all Ambergris sites had nearly constant anthropogenic sound present from development, and road, boat, and air traffic. Anthropogenic impact on Caye Caulker varied in the study area and ranged from high (rank = 4) to no disturbance (rank = 0). We determined Caye Caulker to be a site of low–moderate (rank = 2–3; *x̄* = 2.5; *SE* = 0.30) disturbance with few sources of constant sound production. Our wetland assessments of the BAL effluent lagoon and surrounding areas determined the study site to have low overall human impact (rank = 1–2; *x̄* = 1.8; *SE* = 0.15). Despite being a commercial aquaculture facility, the effluent lagoon is located on the margins of the farm and receives little chronic anthropogenic sound production, has minimal pollution, and the crocodiles rarely come into direct contact with people.

### 3.2 Call structure

We captured and recorded 33 American Crocodiles from May 2015–August 2016; 17 hatchlings, 12 juveniles, 2 sub‐adults, and 2 adults (Boucher, 2017). In total, we analyzed 153 distress calls, a combined 7 calls from 2 individuals were excluded from the analyses as measurements could not be attained due to background noise. American Crocodiles produced multiple distress calls during each recording event but number of calls produced by size class did not differ for 10‐s (*F*_3,29_ = 1.36; *p* = .276), 20‐s (*F*_3,29_ = 1.16; *p* = .340), and 30‐s (*F*_3,29_ = 0.92; *p* = .445) time intervals (Boucher, 2017). Number of calls across all size classes averaged 7.10 (*SE* = 0.24) for 10‐s, 11.70 (*SE* = 0.32) for 20‐s, and 15.39 (*SE* = 0.39) for 30‐s intervals.

**FIGURE 4:**
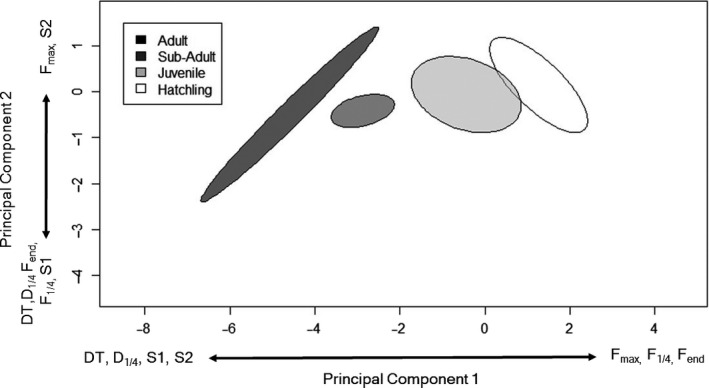
Principal component analysis of parameters for total duration (DT), duration at first quartile (D), maximal frequency (*F*_max_), frequency at first quartile duration (*F*_1/4_), end frequency (*F*_end_), slope of the first quartile (S1), slope of the last three quartiles (S2), and individual size class of American Crocodile distress calls in Belize, 2015–2016. Ordinal ellipse illustrates core size class groupings in relation to call parameters. Call variables on the principal component axes indicate the measured variables driving the separation of individuals by size class

American Crocodile distress calls in Belize have strong frequency modulation and are comprised of multiple harmonics with downsweeping frequencies (Figure 3a). Call structure was consistent across size classes; however, measured call parameters differed. Measured variables differed among all size classes (Wilks, *α* = 0.05; *F*_3,149_ = 16.86, *p* < .001). All acoustic variables differed significantly among size classes for total duration and duration of the first quartile (*H*_3_ = 91.73; *p* < .001), maximal frequency of the fundamental (*H*_3_ = 64.39; *p* < .001), frequency at first quartile (*H*_3_ = 49.46; *p* < .001), ending frequency (*H*_3_ = 32.10; *p* < .001), first quartile slope (*H*_3_ = 78.75; *p* < .001), and slope of the last three quartiles (*H*_3_ = 78.49; *p* < .001) (Table 2). Hatchling calls had the highest frequencies (*F*_max_, *F*_1/4_, *F*_end_) and greatest call modulation (Slope 1, Slope 2), but the shortest total durations. Conversely, adult distress calls exhibited longer total duration and had the lowest frequencies and call modulation. Call parameters allowed for successful discrimination of individuals among size classes but not all parameters had equal success. Temporal variables (DT, *D*_1/4_) differentiated all size classes; however, frequency modulation (Slope 1, Slope 2) differed less and was not effective in discriminating between sub‐adults and adults (Table 2). Frequency parameters had mixed effectiveness of differentiating size classes by acoustic parameters. Results from our principal components analysis complimented result of the one‐way analyses demonstrating separation and grouping of size classes from the measured variables (Figure 4). Cross‐validation through discriminant function analysis demonstrated 82.4% overall success in appropriate size class classification of individuals. We achieved greatest classification accuracy for hatchlings (89.7%), followed by juveniles (81.8%), sub‐adults (70.0%), and adults (40.0%).

**TABLE 2:** Mean and standard error for total duration (DT), duration at first quartile (*D*_1/4_), maximal frequency (*F*_max_), frequency at first quartile duration (*F*_1/4_), end frequency (*F*_end_), slope of the first quartile (Slope 1), and slope of the last three quartiles (Slope 2) for American Crocodile distress calls by size classes, Belize, 2015–2016^a^

Measurement	Age							
	Hatchling		Juvenile		Sub‐adult		Adult	
	*x̄*	*SE*	*x̄*	*SE*	*x̄*	*SE*	*x̄*	*SE*
DT (s)	0.15 C	0.002	0.21 B	0.01	0.36 A	0.01	0.46 A	0.09
*D*_1/4 _(s)	0.04 C	0.001	0.05 B	0.002	0.09 A	0.002	0.11 A	0.02
*F*_max_ (Hz)	1,043.36 A	23.41	823.09 B	36.83	527.40 C	57.06	287.70 D	15.24
*F*_1/4_ (Hz)	674.11 A	19.12	572.70 B	23.61	436.00 B	40.65	211.50 C	15.39
*F*_end_ (Hz)	185.76 A	7.44	166.91 A	5.49	117.56 B	11.31	107.90 B	6.22
Slope 1 (Hz/s)	9,661.22 A	419.65	4,958.91 B	578.54	1,083.85 C	310.30	624.77 C	104.05
Slope 2 (Hz/s)	4,443.30 A	175.63	2,693.41 B	183.17	1,242.05 C	222.40	470.55 C	105.94

^a^ Means within a row followed by the same uppercase letter are not significantly different (*p* > .05).

### 3.3 Site variance

Call parameters varied between Ambergris Caye and aggregated Caye Caulker and BAL sites for hatchling and juvenile American Crocodile distress calls (Table 3). At Ambergris Caye sites, hatchling calls had greater durations (DT, *D*_1/4_) (*H*_1_ = 5.75; *p* = .02) and call modulation for the first slope (Slope 1) (*H*_1_ = 5.78; *p* = .02), but less modulation for the slope of the last three quartiles (Slope 2) (*H*_1_ = 13.04; *p* < .001). Spectral parameters (*F*_max_, *F*_1/4_, *F*_end_) did not differ for hatchling calls between sites (*p* > .05). Juvenile American Crocodile distress calls differed for all parameters with the exception of Slope 1 (*H*_1_ = 0.001; *p* = .98). Juvenile distress calls produced at Ambergris sites had greater call durations (*H*_1_ = 5.29; *p* = .02). However, we found that juvenile call spectral parameters for *F*_max_ (*H*_1_ = 4.63; *p* = .03), *F*_1/4_ (*H*_1_ = 17.05; *p* < .001), and *F*_end_ (*H*_1_ = 4.52; *p* = .03) to be greater at the aggregated Caye Caulker and BAL sites. We also determined frequency modulation of Slope 2 (*H*_1_ = 23.49; *p* < .001) to be greater for the combined Caye Caulker and BAL sites. Total length (TL) of recorded individuals also differed between sites. Hatchlings captured at Ambergris Caye had greater total lengths than those captured at aggregated Caye Caulker and BAL sites (*H*_1_ = 29.47; *p* < .001); however, juvenile TL was greater for Caye Caulker and BAL sites (*H*_1_ = 4.84; *p* < .03).

**TABLE 3:** Mean and standard error of total duration (DT), duration at first quartile (D), maximal frequency (*F*_max_), frequency at first quartile duration (*F*_1/4_), end frequency (*F*_end_), slope of the first quartile (S1), slope of the last three quartiles (S2), and total length (TL) of hatchling and juvenile American crocodiles at Ambergris Caye, Belize Aquaculture Limited (BAL), and Caye Caulker, Belize, 2015 – 2016. Caye Caulker and BAL data were combined to account for low sample size and facilitate analysis

Variable	Hatchling^a^				Juvenile^b^			
	Ambergris		BAL/Caulker		Ambergris		BAL/Caulker	
	*x̄*	*SE*	x̄	*SE*	*x̄*	*SE*	*x̄*	*SE*
DT (s)	0.15 A	0.003	0.14 B	0.00	0.23 a	0.01	0.19 b	0.01
*D*_1/4 _(s)	0.04 A	0.001	0.03 B	0.00	0.06 a	0.002	0.05 b	0.01
*F*_max_ (Hz)	1,044.73 A	23.66	1,035.00 A	86.22	778.37 b	48.35	901.35 a	52.68
*F*_1/4_ (Hz)	655.66 A	19.09	786.45 A	61.93	499.59 b	18.09	700.65 a	44.49
*F*_end_ (Hz)	189.21 A	8.38	164.73 A	12.11	158.69 b	6.04	181.30 a	10.23
Slope 1 (Hz/s)	−10,088.64 A	452.88	−7,057.84 B	762.51	−5,187.05 a	819.04	−4,559.67 a	708.56
Slope 2 (Hz/s)	−4,198.74 B	180.36	−5,932.86 A	343.74	−2,014.86 b	104.94	−3,880.87 a	333.33
TL (cm)	31.06 A	0.17	29.23 B	0.08	73.57 b	2.25	83.00 a	1.67

^a^ Mean of measured variables between columns, followed by the same uppercase letter, is not significantly different for hatchlings between locations (*p* > .05).

^b^ Mean of measured variables between columns, followed by the same lowercase letter, is not significantly different for juveniles between locations (*p* > .05).

### 3.4 Call production

**FIGURE 5:**
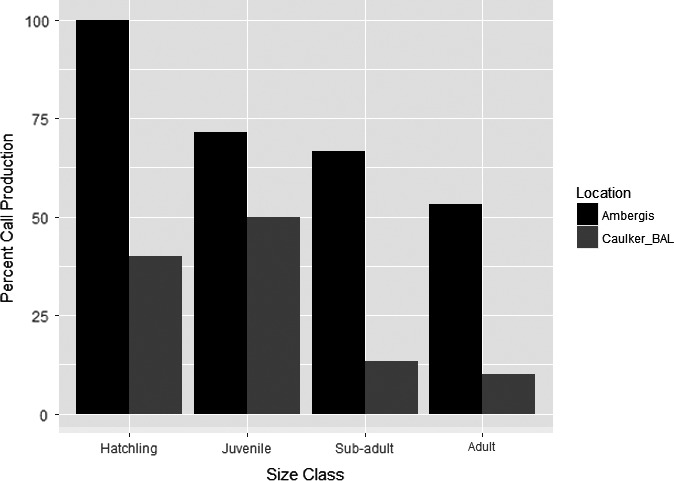
Proportion of American Crocodiles by size class and location that produced distress calls during capture in Belize, 2015–2016

We captured 89 American Crocodiles during the entirety of this project. Of the 89 captured individuals, only 46 (51.7%) produced distress calls. Differences in overall distress call production existed among size classes with hatchlings calling the most and sub‐adults calling the least (χ^2^_3,89_ = 20.44; *p* < .001). We determined that size class call production varied between sites (Figure 5). However, only hatchlings (*p* = .001) and adults (*p* = .03) called more often at Ambergris Caye than at the combined Caye Caulker and BAL sites (Table 4).

**TABLE 4:** Number of American Crocodiles captured by size class, and proportion (% Call) of captured crocodiles that produced distress calls at Ambergris Caye, Belize Aquaculture Limited (BAL), and Caye Caulker, Belize, 2015–2016. Caye Caulker and BAL data were combined to account for low sample size and facilitate analysis

Size class	Overall^a^		Location^b^							
			Ambergris Caye		Caye Caulker		BAL		Caye Caulker/BAL	
	*n*	% Call	*n*	% Call	*n*	% Call	*n*	% Call	*n*	% Call
Hatchling	22	86.4A	17	100.0a	_	_	5	40.0	5	40.0b
Juvenile	24	62.5AB	14	71.4a	7	42.9	3	66.7	10	50.0a
Sub‐adult	18	22.2C	3	66.7a	12	8.3	3	33.3	15	13.3a
Adult	25	36.0BC	15	53.3a	6	0.0	4	25.0	10	10.0b

^a^ Proportion of calls within the overall column, followed by the same uppercase letter, is not significantly different among size classes (*p* > .05).

^b^ Proportion of calls within a column (location), followed by the same lowercase letter, is not significantly different among size classes (*p* > .05).

## 4 DISCUSSION

### 4.1 Call structure

American Crocodile calls are acoustically complex with multiple harmonics. General call structure is the same for all size classes, but call parameters differed among size classes and allowed for successful differentiation of individual's calls to their respective size class. Individually, the measured parameters achieved successful differentiation of larger size classes (sub‐adult and adult) from the smaller size classes (juveniles and hatchlings). When applied together, measured call parameters separated recorded crocodiles into homogeneous groups of their respective size classes. Hatchlings and juveniles emitted calls with higher overall frequencies and call modulation. Adult and sub‐adult American Crocodiles produced distress calls at significantly lower frequencies and have reduced call modulation. There is also a positive correlation between increasing body size and call duration.

Total call duration increased incrementally with body size. This increase in call duration and decrease in call frequency has also been noted for Indian Gharial (*Gavialis gangeticus*) (Bonke, Whitaker, Rodder, & Bohme, 2015). Animal sound production changes as body size increases (Chabert et al., 2015). Lower frequencies are generated by larger crocodiles as the call production mechanisms are larger and produce longer wavelengths (Britton, 2001; Chabert et al., 2015). Crocodylians use low‐frequency sound production to communicate long‐distances, particularly during courtship (Dinets, 2013; Vergne et al., 2009; Vliet, 1989). Hatchling distress calls are higher in frequency and will have limited range as high frequencies do not travel long‐distances as effectively. Post‐hatching American Crocodile hatchlings will remain in cohesive groups monitored closely by the maternal female (Thorbjarnarson, 1989). Call production likely does not need to travel long‐distance to elicit appropriate defense response. However, microhabitat use by juveniles and sub‐adult and adult American Crocodiles differs from hatchlings and yearlings (Thorbjarnarson, 1989). The production of juvenile distress calls at lower frequencies may be beneficial in garnering response from conspecifics that have greater dispersion within a given habitat. Also, larger American Crocodiles occupy more open microhabitats and long‐distance signals can travel unimpeded by the dense shoreline cover that hatchlings prefer.

Differences in call parameters among size classes may reflect differences in behavioral responses. Investigation of distress call information coding demonstrated that crocodylian distress calls produced at higher pitch, with higher frequencies eliciting greater behavioral response from other crocodylians (Staton, 1978; Vergne et al., 2011). Hatchling American Crocodiles produce distress calls at significantly higher frequencies and have greater modulation. During collection of distress calls, we noted that nearby conspecifics reacted more intensely to distress calls from hatchlings compared to the other size classes. Response to distress calls of captured hatchlings was primarily from other hatchlings in the same pod. Size of hatchlings also differed between nest sites and capture of smaller hatchlings from other pods elicited greater response from nonrelated females. Hatchlings also produced distress calls more frequently than the other size classes, which may be another reflection of the significant behavioral response the call elicits. It is likely that the increased behavioral response to hatchling distress calls aids in juvenile survivorship by decreasing predation risk. However, if response to distress calls decreases as frequency and signal modulation decreases it is counterintuitive that sub‐adult and adult American Crocodiles produce distress calls with frequency. Distress calls do not appear to have intrinsic value to individual survivorship as sub‐adult and adult crocodiles have few natural predators (Pooley & Ross, 1989; Staton, 1978). We did not detect any call response from conspecifics when recording distress calls of sub‐adult and adult crocodiles. As such, it makes little sense for crocodiles to produce energetically costly calls that have no perceived benefit. Adult crocodile distress calls, to our best knowledge, have never been reported in such high incidence rates. It may be that the production of distress calls by larger American Crocodiles is indeed the result of anthropogenic activity such as the hydrologic and vegetative modifications we observed in the wetlands, but more likely due to enhanced human presence as evidenced by increased garbage dumping and artificial lighting at night. We further surmise this may be due in part to past or present illegal hunting pressure or harassment in the most modified wetlands.

### 4.2 Call production

Our study is the first to document the frequency of distress call production of American Crocodiles in response to capture. Furthermore, this is the first record of adult American Crocodiles producing distress calls with such regularity. Adult production of distress calls has been reported but described to be a rarity (Staton, 1978). Our results show that a high proportion (36%) of adult American Crocodiles in Belize produce distress calls. This is markedly higher than the production of distress calls for sub‐adults (22%). In particular, we found that the highest proportion of distress calls produced by both adults and sub‐adults came from crocodiles captured on Ambergris Caye study. Ambergris Caye also had the highest proportion of juvenile and hatchling call production. With the exception of hatchlings, we did not find the proportion of call production to be statistically significant for size classes between study sites, which may have been due to our small sample size. This result may be somewhat underestimated as sample size for each size class is low for all sites. However, we noted ecological significance in distress call production. Higher distress call production for hatchlings and adults occurred at the study area with high human impact (rank = 3–4). We found, for juveniles and sub‐adults, lower distress call production at Caye Caulker (rank = 2–3) and BAL (rank = 1–2). Greater call production may be a result of anthropogenic impact in crocodile habitat. As such, production of distress calls could be a metric to determine increased stress levels in American Crocodiles. However, as indicated above, low sample sizes may contribute to this finding, and we suggest further research to investigate the relation among call structure, call production, and anthropogenic disturbance. Moreover, anthropogenic impact has been demonstrated to cause deleterious shifts in wildlife behavior and sound production (Laiolo, 2010).

Anthropogenic sound production causes temporal and frequency shifts in wildlife sound production (Barber, Crooks, & Fristrup, 2010; Laiolo, 2010). Our analyses of call parameters of hatchling and juvenile American Crocodiles demonstrated marked temporal and spectral variances between our sites. Spectral parameters (*F*_max_, *F*_1/4_, *F*_end_) of hatchling distress calls did not differ between sites, but call duration and modulation (Slope 1, Slope 2) did differ. We found call duration (DT, *D*_1/4_) to be greater for hatchling calls recorded at Ambergris Caye; however, TL of hatchlings recorded at Ambergris Caye was also greater ($\bar{X}$ = 31.06; *SE* = 0.17) than that of Caye Caulker and BAL ($\bar{X}$ = 29.23; *SE* = 0.08). It is likely that this variance is a result of increased body size at this site. Hatchling distress calls recorded at Ambergris Caye had higher modulation of Slope 1, and Caye Caulker and BAL sites had higher modulation of Slope 2. Juvenile call parameters varied between sites with the exception of Slope 1. Juvenile American Crocodiles at Ambergris Caye were smaller overall ($\bar{X}$ = 73.57; *SE* = 2.25) than those at the other sites but produced calls of longer duration. This contradicts results from previous study that demonstrate increased body size results in increased call duration (Bonke et al., 2015). We found maximal frequency, *F*_1/4_, *F*_end_, and call modulation of Slope 2 higher at Caye Caulker and BAL sites, which is also contradictory of previous research in which larger crocodiles produce sound at lower frequencies due to increased size of the sound production apparatus (Britton, 2001).

Our results for variance of call parameters between sites complement those of our analysis of distress call production. We observed that the behavioral response of American Crocodiles to distress calls decreased as frequency and modulation decreased, and duration increased. Specifically, American Crocodiles, particularly adults (most likely the mother), responded more intensely in defense of hatchlings by approaching within 7 m of the calling individual. Moreover, previous research on information encoding of juvenile crocodylian calls determined that frequency modulation is the key parameter for behavioral response (Vergne et al., 2012). Higher modulation of the call slope elicited stronger behavioral response from juvenile crocodylians. Slope 2 had greater modulation at our sites of lower anthropogenic impact. Call duration is longer and modulation of Slope 2 less for hatchlings on Ambergris Caye. Ambergris Caye juvenile calls had greater duration, despite smaller body size, and decreased call frequency and modulation. This may indicate that distress calls for hatchling and juvenile American Crocodiles at high impact sites may be structured to be less effective at eliciting a behavioral response. However, it is possible that these modifications are not adaptive and instead may be induced by physiological stress and, or, developmental stress. Further research is required to determine if the differences in call parameters shown here are truly a reflection of anthropogenic impact in the environment. However, in conjunction with increased call production, it is plausible that American Crocodiles may be demonstrating temporal and frequency shifts of distress calls as a result of anthropogenic disturbance.

## 5 CONCLUSIONS

Our results indicate that anthropogenic disturbance may be altering American Crocodile sound production. This could have overarching effects on the behavioral response to distress calls impacting juvenile and hatchling survivorship if defense response is lessened by altered call production. American Crocodiles also use a variety of other acoustic signals to communicate, particularly during courtship, and it is feasible that other calls produced may also be impacted by anthropogenic disturbance. There is ever increasing evidence of human activity and sound production affecting avian, terrestrial, and aquatic wildlife sound production and mitigation of bioacoustic conflict between people and wildlife is emerging. The study of crocodylian bioacoustics is still in its infancy but steps should be taken to further study crocodylian acoustic communication, particularly in response to environmental and acoustic stressors.

## CONFLICT OF INTEREST

None declared.

## AUTHOR CONTRIBUTIONS

**Miriam Boucher:** Conceptualization (equal); data curation (lead); formal analysis (lead); funding acquisition (equal); investigation (lead); methodology (equal); project administration (supporting); resources (supporting); validation (lead); visualization (lead); writing – original draft (lead); writing – review & editing (lead). **Marisa Tellez:** Conceptualization (equal); data curation (supporting); formal analysis (supporting); funding acquisition (supporting); investigation (supporting); methodology (supporting); project administration (supporting); resources (equal); supervision (equal); validation (supporting); visualization (supporting); writing – original draft (supporting); writing – review & editing (supporting). **James T. Anderson:** Conceptualization (equal); Data curation (supporting); formal analysis (supporting); funding acquisition (equal); investigation (supporting); methodology (equal); project administration (lead); resources (equal); supervision (equal); validation (supporting); visualization (supporting); writing – original draft (supporting); writing – review & editing (supporting).

## Data Availability

Data files are available online at Dryad Digital Repository (https://doi.org/10.5061/dryad.q2bvq83gp).
